# Increasing the Chances of Natural Conception: Opinion Statement from the the Brazilian Federation of Gynecology and Obstetrics Associations - FEBRASGO - Committee of Gynecological Endocrinology

**DOI:** 10.1055/s-0039-1677838

**Published:** 2019-02-15

**Authors:** Bruno Ramalho de Carvalho, Ionara Diniz Evangelista Santos Barcelos, Sebastião Freitas de Medeiros, Cristina Laguna Benetti-Pinto, Daniela Angerame Yela, Andrea Prestes Nácul, Gustavo Arantes Rosa Maciel, José Maria Soares Júnior, Ana Carolina Japur de Sá Rosa e Silva, Laura Olinda Bregieiro Fernandes Costa

**Affiliations:** 1 BONVENA - Medicina Reprodutiva, Brasília, DF, Brazil; 2 Department of Gynecology and Obstetrics, Universidade Estadual do Oeste do Paraná, Cascavel, PR, Brazil; 3 Department of Gynecology and Obstetrics, Faculdade de Medicina, Universidade Federal do Mato Grosso, Cuiabá, MS, Brazil; 4 Department of Tocogynecology, Faculdade de Ciências Médicas, Universidade Estadual de Campinas, Campinas, SP, Brazil; 5 Hospital Fêmina, Grupo Hospitalar Conceição, Porto Alegre, RS, Brazil; 6 Department of Gynecology and Obstetrics, Faculdade de Medicina de São Paulo, Universidade de São Paulo, São Paulo, SP, Brazil; 7 Department of Gynecology and Obstetrics, Faculdade de Medicina de Ribeirão Preto, Universidade de São Paulo, Ribeirão Preto, SP, Brazil; 8 Faculty of Medical Sciences, Universidade de Pernambuco, Recife, PE, Brazil

**Keywords:** natural fertility, fertility determinants, fecundability, reproductive period, preconception care, fertilidade natural, determinantes de fertilidade, fecundabilidade, período reprodutivo, cuidados pré-concepcionais

## Abstract

Considering that myths and misconceptions regarding natural procreation spread rapidly in the era of easy access to information and to social networks, adequate counseling about natural fertility and spontaneous conception should be encouraged in any kind of health assistance. Despite the fact that there is no strong-powered evidence about any of the aspects related to natural fertility, literature on how to increase the chances of a spontaneous pregnancy is available. In the present article, the Brazilian Federation of Gynecology and Obstetrics Associations (FEBRASGO, in the Portuguese acronym) Committee on Endocrine Gynecology provides suggestions to optimize counseling for non-infertile people attempting spontaneous conception.

## Resumo

Uma vez que mitos e equívocos sobre a procriação natural se espalham rapidamente na era do fácil acesso à informação e às redes sociais, o aconselhamento adequado sobre a fertilidade natural e a concepção espontânea deve ser encorajado em qualquer tipo de assistência à saúde. Apesar do fato de não haver evidências fortes sobre qualquer dos aspectos relacionados à fertilidade natural, existe literatura sobre como aumentar as chances de uma gravidez espontânea. No presente artigo, a Comissão Nacional de Ginecologia Endócrina da Federação Brasileira das Associações de Ginecologia e Obstetrícia (FEBRASGO) oferece sugestões para otimizar o aconselhamento a pessoas que tentam a concepção espontânea, na ausência do diagnóstico de infertilidade.

## Introduction

Doctors in any specialty and any other health professionals must be encouraged to provide adequate counseling about sexual and lifestyle habits that may be related to natural fertility and spontaneous conception.

There are many myths and misinterpretations of information regarding the optimization of the chances of natural procreation. The easy access to information and to social networks today allows distorted concepts to spread with enormous rapidity, causing a commotion of immeasurable impact, and even confusing health professionals.

In fact, to date, there are no strong-powered studies in any aspect related to natural fertility, so the current knowledge is insufficient to support definitive recommendations. However, the available literature, based on consensual expert opinion, provides suggestions for counseling patients about how they should increase the chances of having a spontaneous pregnancy.

In the present article, we provide subsidies to overthrow myths and misconceptions in the daily routine of reproductive counseling, based on the best evidence available.

## The Impact of Female Age

Advances in knowledge and better access to information on healthy living habits cause a great confusion between the concepts of youth and joviality. From the dissociation of those concepts, appears the idea that people at the age of 40 years old today are as healthy as those who were 20 or 30 years old in the past—or even healthier. Unfortunately, regarding female fertility, time exerts many changes on reproductive function; thus, although a woman may look and feel good in her 40s, her fertility is not the same as that of a woman in her 30s.

The statistics are not unanimous, but recent data indicate that cumulative pregnancy rates after 12 cycles of attempt range from almost 80% at the age between 25 and 27 years old to 55% at the age between 40 and 45 years old.^1^ As it happens, pregnancy rates have been invariable, at least in the last three decades.^2^

There is a plausible biological explanation for the decline in human fecundability with increasing female age: the decrease in the quantity, in the quality, and in the reproductive potential of the eggs. At birth, the number of eggs in both ovaries is in the millions, but at the beginning of puberty, women have only hundreds of thousands, which will be consumed over the 400 or so ovulatory cycles in their reproductive lives. It is believed that around the age of 38 years old, there is a significant acceleration of egg consumption, and although much is discussed about the 35-year-old frontier, the interval between 37 and 38 years old is probably the most significant in terms of loss of female reproductive potential.^3^ ^4^

According to the mathematical model published by Habbema et al (2015),^5^ the chances of a couple to realize the desired family size can be estimated from the age of the woman when initiating pregnancy attempts. It predicted that couples with a desire to have only one child would have a 90% chance of spontaneous pregnancy if attempts were initiated until the woman reached 32 years old. However, when the goal of the couple was to naturally conceive two or three children, the woman should initiate attempts at the ages of 27 and 23 years old, respectively, at the risk of failing to reach the intended offspring.^5^

Therefore, considering that the probability of conception is highly dependent on maternal age, the impact of modern lifestyle and delaying childbearing can decrease the likelihood of successful spontaneous conception.

## The Impact of Male Age

As it has been observed for women, there is a tendency for increased paternal age in the reproductive function in developed countries, and more attention has been paid to the effects of age on this function.^6^ The association between male age and reproductive potential for natural conception is little known, but is becoming an emerging issue.

The mathematical model published by Dunson et al (2002)^7^ highlighted the negative impact of male age on the chances of spontaneous pregnancy when women ≥ 35 years old had sexual partners at least 5 years older, but there is no doubt that in these cases the age of the woman becomes a confounding factor. In parallel, in a recently published study from the USA, male age adjusted for female age was not associated with fecundability, although the number of men > 45 years old was small.^1^

With the evidence available to date, there is no data to support or rule out such an association, but there are clear indications of the negative effect of advanced paternal age on seminal parameters,^8^ ^9^ such as the amount of produced sperm cells and their motility,^10^ and an increased risk of genetic, neurological, and psychiatric diseases in the offspring is also observed.^8^ A recent meta-analysis of 81 studies found some evidence of the association between paternal age and problems such as autism, autism spectrum disorders, schizophrenia, stillbirth, birth defects, and aneuploidies.^11^

## The Mathematical Model for Fertility Window

**Fig. 1:**
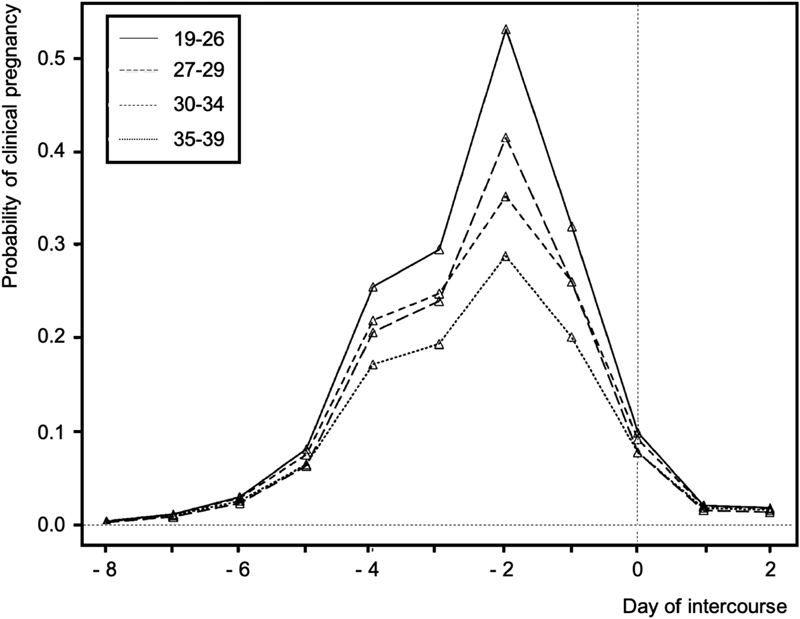
Probability of clinical pregnancy following intercourse on a given day in relation to ovulation, for women of average fertility, aged 19–26, 27–29, 30–34 and 35–39 years old (data were drawn from the European Study of Daily Fecundability, including 433 pregnancies), adjusted for the age of the male partner (Dunson et al, 2002).^7^ This figure has been reprinted with permission from Oxford University Press on behalf of the Brazilian Federation of Gynecology and Obstetrics Associations (FEBRASGO, in the Portuguese acronym) Committee on Endocrine Gynecology, for publication on FEBRASGO's official journal, RBGO Gynecology and Obstetrics.

It is easy to understand why people believe in maximum female fertility on the day of ovulation, and it is still very common to see people holding intercourses for a day or two beyond that date in the belief that there is still fertility. However, with the support of the American Society for Reproductive Medicine (ASRM) and of the Society for Reproductive Endocrinology and Infertility,^12^ mathematical models define the fertile period as a window that begins five days before ovulation and ends on the day it happens. Furthermore, it is assumed that the greater chance of natural pregnancy is associated with coitus occurring two days before ovulation, and this chance decreases as coitus distances from this point (Fig. 1).^7^ Thus, the chances of natural conception are greatest over a period of 6 days ending on the day of ovulation; on the other hand, although one cannot speak of impossibility, chances of conception are very low outside this interval.

Signs of ovulation can also be used for counseling on natural fertility, especially when it is not possible to identify the fertile period based on the duration of the cycle or when the comprehension of the mathematical model is not reached by the couple. The signs of easier identification are the filamentous mucus or type 4 mucus—elastic, slippery and transparent—, and the elevation of basal temperature, but both behave as expected in only ∼ 60% of the cycles^13^ ^14^ and, therefore, will not always be effective in predicting the fertile window.

Finally, it should be noted that the fertile period does not change with the advancement of the age of the woman; the chance of conception is what diminishes with aging. Thus, with isolated intercourse 2 days before ovulation, the chance of pregnancy in a woman between the ages of 35 and 39 years old corresponds to approximately half the chance of a woman < 26 years old.^7^

## The Common Practice of Coitus Every Other Day

The information that one could have a greater chance of conception if the practice of coitus occurs every other day is very common, considering a misperception that frequent ejaculations could lead to a diminished number of sperm count. However, a retrospective study that analyzed nearly 10,000 semen specimens observed that, in men with normal semen quality, sperm concentrations and motility remain normal, even with daily ejaculation. Interestingly, in men with oligozoospermia, sperm concentration and motility may be highest with daily ejaculation.^15^ In one study involving 221 presumably fertile couples planning to conceive, the highest cycle fecundability (37% per cycle) was associated with daily intercourse.^16^

In spite of the varied quality of existing studies, evidence suggests that reducing the ejaculatory abstinence period may positively influence semen quality based on a consistent trend toward an increase in the percentage of motile, progressively motile and rapid spermatozoa with shorter abstinence periods.^17^ Therefore, shortening the abstinence period may be a potential strategy to improve sperm quality,^18^ while longer intervals are associated with lower pregnancy rates. Although evidence suggests that daily intercourse may confer a slight advantage, specific recommendations regarding the frequency of intercourse may unnecessarily induce stress. Couples should be advised that the optimal frequency of intercourse is best defined by their own preference.^12^

## The Positions Adopted for Intercourse

The positions adopted for intercourse or how women remain after coitus have no association with improved chances of pregnancy. There is no need for any postcoital routine, and although many women believe that remaining in a supine position for an interval period after intercourse facilitates sperm transport and prevents leakage of semen from the vagina, this belief has no scientific foundation.^12^

Some studies have demonstrated that rapid sperm transport through the female genital tract is passive and is provided by uterine contractions. Using hysterosalpingoscintigraphy, rapid sperm transport was studied by placing technetium-labeled albumin macrospheres of sperm size at the external cervical os. The ascension of the macrospheres occurred immediately following deposition at the external os of the cervix. As early as 1 minute thereafter, the macrospheres had reached the intramural and isthmical part of the tube.^19^ Another study has documented that within 15 minutes after the coitus, a constant level of sperm exists in the oviduct.^20^ Still regarding intercourse, the presence or absence of female orgasm does not seem to alter the probability of conception.^12^

## The Use of Intimate Lubricants

Many couples trying to conceive require a coital lubricant when suffering from vaginal dryness or discomfort during intercourse. However, there is much uncertainty regarding the ideal fertility-preserving coital lubricant.^21^ Couples are faced with a great number of options and are often worried whether the use of these substances might compromise their chance to achieve pregnancy.^22^ Multiple lubricants have been shown in multiple studies to adversely affect sperm motility at a variety of concentrations.^23^ ^24^ ^25^ ^26^ ^27^ One study showed sperm to be immotile after 15 minutes of exposure to these lubricants.^24^ Since vaginal lubricants have been shown to affect sperm motility in vitro, it is possible that lubricant use during intercourse may negatively affect natural fertility by inhibiting fertilization.^4^ ^21^ These findings reaffirm the notion that not all coital lubricants and oils are alike, and that individual lubricants and oils must be carefully evaluated regarding their effects on fertility.

A recent study has addressed this issue and evaluated the effects of coital lubricants and oils on sperm motility. Changes in total and progressive motility were variable for sperm incubated under different oils. Both total and progressive sperm motility slightly decreased after exposure to canola and baby oils; however, this decrease was not significant. After the initial decrease, sperm motility remained stable at high levels within up to 60 minutes of incubation. This slight drop in motility is understandable and can be attributed to the adjustment of sperm to a new microenvironment. It was not observed a negative impact of canola and baby oils on sperm motility. This result was attributed to the presence of nontoxic ingredients in these oils. Sesame and mustard oils were incorporated into the study due to the lack of information regarding their effects on sperm. Sesame oil showed an immediate drastic decline in both total and progressive sperm motility within 5 minutes of incubation. Sperm motility continued to decline nonsignificantly over the course of incubation under sesame oil. In contrast, exposure of sperm to mustard oil initiated hyperactive motility and the sperm remained hyperactive during the entire incubation period without any decline in motility.^22^

The coital lubricants and oils that were found to have negative effects on sperm motility should be avoided. Therefore, when suffering from vaginal dryness or discomfort during intercourse, canola, baby or mustard oil should be preferred, as they appear to not affect significantly sperm motility.

## Is it Possible to Define the Gender of the Baby on the Day of Ovulation?

Common popular belief states that intercourse closest to the day of ovulation would favor the conception of boys, and that intercourse more distant from the time of ovulation would favor the conception of girls. Even though this is such a strong belief, limited and controversial evidence is currently available.

A recent study indicates that when comprehensive instruction is provided, the gender of a child can be preselected with a high degree of confidence by timing coitus, using the Post-Peak approach of Billings Method for males and Pre-Peak for females.^28^ Another study evidenced that the gender ratio favored males when intercourse preceded ovulation/fertilization by two days or longer. While this association was statistically significant, the number of pregnancies involved was too small to conclude that the relationship is real.^29^

On the other hand, a prospective study of 33 pregnancies using the rise in luteinizing hormone in the early morning urine, the peak cervical mucus symptom, and the shift in basal body temperature as indicators of ovulation clearly refuted the theory that intercourse close to ovulation favors the conception of males.^30^

For routine application, timing sexual intercourse to determine the gender of the baby may not be part of the counseling strategy,^16^ ^31^ since there is no strong evidence of a real connection. In reality, there is a small number of studies, most of them performed more than 15 years ago, in small groups of patients, and showing contradictory results that do not support a consensual position.

## Diet and Female Fertility

There is scarce good scientific data relating diet and fertility, and the real benefits of vegetarian or low-fat diets, or of the intake of multivitamins, antioxidants or herbal supplementation, are not consensual.^12^

Among specific micronutrients, folic acid and vitamin D seem to be the most studied, but robust evidence in the literature is still lacking.^32^ Observational studies suggested an association between folic acid intake and a reduced risk of spontaneous miscarriage among women attempting pregnancy or during its early weeks, especially if the intake levels were above those recommended for the prophylaxis of neural tube defects.^33^ ^34^ ^35^ Also, folic acid consumption appeared to be related to a reduction in the risk of annovulation^36^ and should be related to a shorter time to pregnancy.^37^ Vitamin D status or its supplementation, by the way, is poorly associated with annovulation, probability of conception, time to pregnancy, or pregnancy losses.^38^ ^39^ ^40^ ^41^

In a large preconception cohort, trans fat intake was associated with reduced fecundity, and consumption of omega-3 polyunsaturated fatty acids was associated with higher fecundity in the absence of fish oil supplementation; in fact, these associations were found only in American women, and these effects were not found in Danish women, although the intake amount among Danish women varied from low to rare.^42^

In a cohort of 17,544 women without a history of infertility who were followed-up for 8 years, those who had the greatest intake of protein from vegetables, full-fat dairy food, iron, and monounsaturated fats during the preconception period had significantly higher chances of becoming pregnant when compared with women with different diet patterns, controlling for age, body mass index (BMI), alcohol and coffee consumption, smoking, and use of oral contraceptives.^43^

A possible decrease in the risk of anovulatory infertility was observed in the regular intake of multivitamin supplements,^36^ the replacement of animal protein for vegetable sources of protein,^44^ lower carbohydrate intake and dietary glycemic load,^45^ and preconception intake of high amounts of whole grains.^46^

Finally, dietary habits have also been investigated for reproductive effects. Excessive caffeine consumption (over 5 cups of coffee per day) may decrease fertility,^12^ but the 8-year follow-up of 18,555 married women did not support a causal relation between alcohol or caffeine intake and decreased fertility. In the same cohort, the association between soft drinks and anovulatory infertility could not be related to caffeine or sugar content.^47^

In summary, current limited evidence on the reproductive effects of diet supports benefits from folic acid supplementation before and during early pregnancy, low dietary glycemic load and consumption of high amounts of whole grains, higher intake of omega-3 polyunsaturated fatty acids, lower intake of trans fatty acid, and fish as a source of animal protein.

## Diet and Male Fertility

Since the early 1980s, several nutrients and components have been considered as possible determinants of sperm function, fertility, or of normal function of the reproductive system.^48^ Accumulating evidence from human in vitro and animal studies indicates that male obesity and some components of the diet may play an important role in modulating spermatogenesis, sperm maturation, and fertilizing ability. For example, male obesity has been related to impaired fertility because of its effect on the molecular and physical structure of sperm.^49^ ^50^ ^51^

Moreover, several components of the diet that have been associated with an increased risk of obesity, of insulin resistance, and of diabetes have also been related to low sperm quality or function in animal models. For example, diets rich in trans-fatty acids, saturated fats^52^ or cholesterol^53^ have been associated with testicular disruption, involving impairments in spermatogenesis, potentially affecting male fertility and the offspring in rats and rabbits. Testicular metabolic alterations induced by high-calorie diets may also lead to mitochondrial dysfunction, which is closely associated to reactive oxygen species (ROS) overproduction and oxidative stress. Reactive oxygen species easily targets spermatozoa DNA and lipids, contributing to decreased sperm quality.^54^

On this particular subject, we have very low-quality randomized controlled trials, conducted in small samples of participants, investigating the effect of specific nutrients and of nutritional supplements on male infertility.^55^ However, in spite of the lack of proper evidence about the role of diet in sperm parameters and about the effectiveness of supplements to combat male infertility, there has been an invasion of integrative dietary products in the last two decades in some assisted reproductive technology (ART) clinics. Unfortunately, the safety of these dietary supplements has not been tested, and the dangers for the user population are unknown.^56^

Although based on epidemiological observational design studies, which limits the ability to determine causality between the intake of food and nutrients and the parameters of semen quality and fecundability, a recent and extensive review indicated that healthy diets rich in some nutrients such as omega-3 fatty acids, some antioxidants (vitamin E, vitamin C, β-carotene, selenium, zinc, cryptoxanthin, and lycopene), other vitamins (vitamin D and folate), and low in saturated fatty acids and trans-fatty acids were inversely associated with low semen quality parameters.^56^

Fish, shellfish and seafood, poultry, cereals, vegetables and fruits, low-fat dairy and skimmed milk were positively associated with several sperm quality parameters. However, diets rich in processed meat, soy foods, potatoes, full-fat dairy and total dairy products, cheese, coffee, alcohol, sugar-sweetened beverages and sweets have been detrimentally associated with the quality of semen in some studies.

In summary, as far as fecundability is concerned, a high intake of alcohol, caffeine, red meat and processed meat by males has a negative influence on the chances of pregnancy or on the fertilization rates in their partners.^56^ ^57^ On the other hand, regular consumption of fish and seafood, poultry, cereals, vegetables and fruits, and low-fat dairy products can improve seminal parameters.

## The Impact of Smoking

Smoking has a negative impact on the overall health of men and women, which may include the reproductive function. Scientific research on the subject suggests that smoking may lead to a decline in the natural fertility of women and men.^58^ ^59^ ^60^ ^61^

The literature demonstrates decreased chances of pregnancy, increased spontaneous miscarriage rates, and earlier onset of menopause in smoking women when compared with non-smokers.^12^ ^62^ ^63^ ^64^ ^65^

In smoking men, semen quality maybe impaired by a decrease in sperm count, an increase in malformed gametes, and possible aggression to sperm DNA.^12^ ^63^ ^64^ ^65^

It must be stressed that there are no large-scale, randomized, clinical trials examining the effect of cigarette smoking on fertility. Even though scientific unanimity is not obtained, the data accumulated to date support the value of the preventive approach to infertility, and for this reason, discourage smoking or exposure to smoking for men and women attempting pregnancy,^66^ even when ART is recommended.^67^

## Tests for Measuring Fertility among Non-infertile Women

The cumulative probability of conception and the relative probability of conception in a given menstrual cycle (fecundability) were prospectively evaluated in 750 women without a history of infertility; low serum antimüllerian hormone (AMH) or high follicle-stimulating hormone (FSH) levels were not associated with reduced fertility in at least the following 12 cycles of attempt, even after controlling for age, race, BMI, smoking status, and recent use of hormonal contraceptives.^68^

In a recent multicenter cross-sectional study, no differences in serum AMH and antral follicle count (AFC) were found between healthy normo-ovulatory women with unexplained infertility and women not seeking treatment for fertility, even after controlling for personal confounders or study site.^69^

Finally, other recent studies had already demonstrated that AFC and AMH levels did not differ between fertile and infertile women of the same age,^70^ ^71^ and that spontaneous pregnancy may occur even in women presenting low AMH or AFC levels, or high FSH levels.^72^ ^73^

In the light of the current knowledge, ovarian reserve tests cannot be parameters for inferences about the reproductive potential in the medium or long term. Thus, they cannot be decisive for women to postpone maternity or not, and there is no indication to include ovarian reserve evaluation as a clinical routine for young women attempting to get pregnant, even if they had never been mothers, since they may not predict natural fertility alone, neither currently nor in the future.

There is still much knowledge to be obtained regarding natural fertility. An increasing amount of data have been associating the exposure to environmental pollutants, toxicants, marijuana and other recreational drugs, and job-related exposures (microwaves, heat, pesticides and other chemicals, for example) with reduced fecundability and infertility.^12^

Finally, observational studies have suggested that stress, frequent in the present days, is associated with infertility. However, no clinical trial has demonstrated definitely that reducing stress prior to infertility treatment improves pregnancy rates. Attempts to isolate single causal links between stress and infertility have not yet been successful due to their multifaceted etiologies.^74^
